# Simvastatin Attenuates Abdominal Aortic Aneurysm Formation Favoured by Lack of Nrf2 Transcriptional Activity

**DOI:** 10.1155/2020/6340190

**Published:** 2020-06-16

**Authors:** Aleksandra Kopacz, Ewa Werner, Anna Grochot-Przęczek, Damian Klóska, Karolina Hajduk, Christoph Neumayer, Alicja Józkowicz, Aleksandra Piechota-Polanczyk

**Affiliations:** ^1^Department of Medical Biotechnology, Faculty of Biochemistry, Biophysics and Biotechnology, Jagiellonian University, Krakow, Poland; ^2^Department of Animal Reproduction and Anatomy, Faculty of Animal Science, University of Agriculture, Krakow, Poland; ^3^Department of Surgery, Division of Vascular Surgery, Medical University of Vienna, Austria

## Abstract

Surgical intervention is currently the only option for an abdominal aortic aneurysm (AAA), preventing its rupture and sudden death of a patient. Therefore, it is crucial to determine the pathogenic mechanisms of this disease for the development of effective pharmacological therapies. Oxidative stress is said to be one of the pivotal factors in the pathogenesis of AAAs. Thus, we aimed to evaluate the significance of nuclear factor erythroid 2-related factor 2 (Nrf2) transcriptional activity in the development of AAA and to verify if simvastatin, administered as pre- and cotreatment, may counteract this structural malformation. Experiments were performed on mice with inhibited transcriptional activity of Nrf2 (tKO) and wild-type (WT) counterparts. We used a model of angiotensin II- (AngII-) induced AAA, combined with a fat-enriched diet. Mice were administered with AngII or saline for up to 28 days *via* osmotic minipumps. Simvastatin administration was started 7 days before the osmotic pump placement and then continued until the end of the experiment. We found that Nrf2 inactivation increased the risk of development and rupture of AAA. Importantly, these effects were reversed by simvastatin in tKO mice, but not in WT. The abrupt blood pressure rise induced by AngII was mitigated in simvastatin-treated animals regardless of the genotype. Simvastatin-affected parameters that differed between the healthy structure of the aorta and aneurysmal tissue included immune cell infiltration of the aortic wall, VCAM1 mRNA and protein level, extracellular matrix degradation, TGF-*β*1 mRNA level, and ERK phosphorylation, but neither oxidative stress nor the level of Angiotensin II Type 1 Receptor (AT1R). Taken together, the inhibition of Nrf2 transcriptional activity facilitates AAA formation in mice, which can be prevented by simvastatin. It suggests that statin treatment of patients with hypercholesterolemia might have not only a beneficial effect in terms of controlling atherosclerosis but also potential AAA prevention.

## 1. Introduction

An aortic aneurysm is a permanent dilatation of the aorta with structural malformations within all three layers of the vascular wall. It develops most frequently in the infrarenal aorta and is called then an abdominal aortic aneurysm (AAA). Pathophysiology of AAA formation, although still not entirely elucidated, involves progressive upregulation of proteolytic pathways, apoptosis of vascular smooth muscle cells (VSMCs), oxidative stress, and inflammation, as well as collagen deposition which compensates elastin degradation [1–3]. Most of these processes are regulated by Nrf2 (nuclear factor (erythroid-derived 2)-like 2), a stress-responsive transcription factor, which activates the expression of cytoprotective, antioxidant, and detoxification genes [4, 5]. Nrf2-dependent pathways were also shown to counteract cardiovascular disorders by the maintenance of VSMC contractile phenotype, regulation of vascular tone, reduction of lipid accumulation, and macrophage influx into the media layer as well as inhibition of vascular calcification [5], which are all hallmarks of AAA [2, 3]. However, the significance of Nrf2 in AAA formation has not been addressed yet.

Statins, inhibitors of 3-hydroxy-3-methylglutaryl-coenzyme A (HMG-CoA) reductase, are well-known lipid-lowering drugs, which possess potent antioxidant and anti-inflammatory properties [6]. Throughout the years, AAA was considered to develop on the atherosclerotic background. More recent data suggest, however, that AAA and atherosclerosis are different disease entities, though they share common risk factors [2]. Statins are drugs of choice often administered to AAA patients due to the accompanying hypercholesterolemia [7]. Although many studies demonstrated the beneficial effect of statins on AAA growth, the more extensive clinical trials did not confirm these findings [2, 8]. Therefore, the potential use of statins in AAA is still a matter of debate, particularly in the context of preventive therapy.

Given the potential role of Nrf2 and statins in AAA development, we investigated if abrogation of Nrf2 transcriptional activity influences the AAA occurrence in mice and, if so, whether simvastatin treatment before and during aneurysm formation modulates its onset. We found that transcriptional knockout of Nrf2 in mice increased the risk of AAA formation and rupture compared to wild-type animals. It was accompanied by high aortic inflammation and increased activity of gelatinases. Remarkably, AAA occurrence in the Nrf2-defective mice was attenuated by simvastatin administration.

## 2. Methods

### 2.1. Animals

Experiments were performed in C57BL/6J mice with the functional Nrf2 (wild-type (WT)) or with the transcriptionally inactive form of Nrf2 (transcriptional knockout (tKO)). Mice were generated as described previously [9] and kindly provided by Prof. Antonio Cuadrado (Universidad Autonoma de Madrid, Spain). In these mice, a sequence coding for carboxyl amino acid residues of Nrf2 (including DNA binding domain) was replaced by LacZ gene. It results in the presence of fusion protein Nrf2-LacZ, consisting of remaining N-terminal 301 amino acids of Nrf2 being linked to *β*-gal [10]. Upon arrival at our facility, mice have been bred to C57BL/6J every ten generations. Six-month-old male mice of verified genotype were used (4–7 animals per group at each time point). The genotype was verified prior to the experiment by DNA analysis and after the experiment by *NFE2L2* mRNA assessment (Fig. S1A-B). The animals were maintained under specific pathogen-free conditions in the individually ventilated cages (14/10 h light/dark cycle at a temperature of 22 ± 2°C) and were provided with a fat-enriched diet (25% fat) and water *ad libitum.* All experimental procedures were approved by the Second Local Ethics Committee for Animal Experiments in Krakow (No. 74/2016) and performed in accordance with the guidelines from Directive 2010/63/EU of the European Parliament on the protection of animals used for scientific purposes.

### 2.2. Experimental Groups

WT and tKO mice were divided randomly into the following groups: (1) sham (saline, *n* = 8), (2) angiotensin II (1000 ng/kg/min) (AngII group, *n* = 10 or 14), (3) simvastatin (20 mg/kg/day, *i.g.*)+saline (Sim group, *n* = 12), and (4) simvastatin (20 mg/kg/day, *i.g.*)+angiotensin II (1000 ng/kg/min) (Sim+AngII group, *n* = 13).

### 2.3. Development of Abdominal Aortic Aneurysm

Mice were infused *via* osmotic minipumps (Alzet 2004) with angiotensin II (1000 ng/kg/min in saline; Sigma-Aldrich) or saline (sham group) for 28 days. Osmotic pumps were placed subcutaneously under isoflurane (Aerrane, Baxter; 5% *v* : *v* in the air) anaesthesia. Simvastatin (20 mg/kg/day in saline; Sigma-Aldrich) was administrated each morning *via* intragastric gavage (*i.g.*) for 7 consecutive days before the placement of the osmotic pump and also during AngII infusion. Simvastatin action was confirmed by an increase in HMG-CoA reductase mRNA (*HMGCR*) [11] both in the liver and in the abdominal aorta (Fig. S1C-D). The scheme of the experiment is presented in Fig. S2.

The following parameters were monitored every seven days: body weight, blood pressure (BP), aortic diameter, and appearance by ultrasonography (USG) (Fig. S2). BP change after osmotic pump placement was additionally measured on day 2. The mice were sacrificed on day 14 or 28 by overdosing of carbon dioxide; the blood and aortas were collected for further biochemical analysis. The aortas were cleaned from the adjacent connective tissue and fat, photographed on a scale ruler. They were further preserved in OCT freezing medium for histological staining or in RNAlater (Sigma-Aldrich) for gene expression analysis. In this study, all the analyses were performed on the abdominal part of the aorta.

### 2.4. Blood Pressure Monitoring

All mice underwent noninvasive blood pressure measurement by tail-cuff plethysmography (BP-2000 series II, Visitech Systems) which was preceded by a period of adaptation. The measurements were performed by one person to decrease any bias. The results were presented as a percentage change compared to day 0 (after adaptation time and before the administration of any drugs). This way of data presentation was chosen to plot the impact of administered compounds better and to minimise the influence of phenotypic and age-related changes between littermates. Importantly, no difference in mean blood pressure between genotypes was observed at day 0.

### 2.5. *In Vivo* Ultrasound Imaging of Abdominal Aorta and Analysis

Changes in the abdominal aorta diameter were monitored following [12], using the high-frequency ultrasound imaging system (Vevo 2100, FUJIFILM VisualSonics). Two-dimensional (B-mode) imaging using a 22-55 MHz linear-array transducer (MS550D) synchronised to the electrocardiographic signal was done. The animals were placed in a supine position on a heated table under inhalation anaesthesia with isoflurane (1.5-2%). The abdominal cavity was shaved, and a prewarmed ultrasound gel was applied to the area of interest. Longitudinal images of the suprarenal and infrarenal aorta and transverse images at the level of the abdominal aorta between the diaphragm and the outlet of the left renal artery were acquired in the B-mode and M-mode to assess the maximum cross-sectional diameter (during diastole) and aortic area in real-time for each mouse at each time point. Each animal was inspected in both B-mode and M-mode when analysing the north-south axis (longitudinal) and in B-mode for the west-east (transversal) axis. B-mode images were taken between the diaphragm and the mesenteric artery. At the M-mode, the abdominal aorta was visualised between the diaphragm and iliac arteries. The aorta was circled using enough number of markers to reflect its shape. To assess the aortic diameter and area, three consecutive images of the aorta were marked. Using the software tool for aortic diameter measurement, the beginning and the end of the line segment were pointed, and the aortic diameter in millimetres (mm) was measured by the software. Each time, three line segments between the diameter and mesenteric arteries were analysed to provide a mean value of the diameter. The scheme of analysis is shown in the supplementary materials (Fig. S3), given that the most reproducible results, and burdened with the lowest error, were obtained from M-mode measurements. Thus, results analysed this way were included in the manuscript. Similar to blood pressure measurements, the data is presented as the change of day 0 in order to outline the general trend of changes upon receiving compounds. Importantly, no difference in the mean aortic diameter between genotypes was observed at day 0.

### 2.6. Blood Cell Count

Right after euthanasia, approximately 1 mL of blood was collected from retroorbital sinus to a tube coated with EDTA. Blood cell count was analysed using an ABC Vet Haematology Analyzer (Horiba).

### 2.7. Total RNA Isolation, Reverse Transcription, and Quantitative PCR

RNA from 5 mm fragment of the abdominal aortic tissue was extracted with the RNeasy Mini Kit (Qiagen) according to the manufacturer's instructions. cDNA was synthesised using a High-Capacity cDNA Reverse Transcription Kit (Thermo Fisher Scientific). RT-qPCR was conducted on Step-One Plus Real-Time PCR Systems using a Power SYBR® Green PCR Master Mix according to the manufacturer's instructions (Thermo Fisher Scientific). Primer sequences are gathered in Table S1. Eukaryotic mouse translation elongation factor 2 (*eEF2*) was used as a reference gene. Relative gene expression was calculated using the *ΔΔ*Ct method.

### 2.8. Assessment of Redox Status of the Aorta

Detection of reactive oxygen species (ROS) was performed with CellROX Deep Red Reagent (Thermo Fisher Scientific) and analysed using a meta laser scanning confocal microscope (LSM-880; Carl Zeiss).

### 2.9. Histological and Immunofluorescent Analysis

Immunofluorescent stainings were done in frozen 20 *μ*m (collagens I and III, phospho-ERK), 30 *μ*m (AT1R), or 40 *μ*m (CD45, CD64) specimens of the abdominal aorta. Elastin was stained on 5 *μ*m tissue slices using the Verhoeff-van Gieson method available on IHCworld protocols website. Samples were analysed under a light microscope (Nikon) with NIS elements BR software (Canon) at magnifications of 100x and 200x.

Immunofluorescent stainings were performed on frozen tissue slides. Samples were fixed with methanol (for AT1R and phospho-ERK) or ice-cold acetone (for collagens I and III and CD45/CD64) and blocked in 10% goat serum (GS) with 0.05% Tween-20 for 1 h at room temperature (RT). After washing in PBS, samples were incubated overnight (4°C) with rabbit anti-AT1R polyclonal IgG antibodies (dilution 1 : 100; Abcam), rabbit anti-collagen I or collagen III IgG polyclonal antibodies (dilution 1 : 250; Abcam), rabbit anti-phospho-ERK1/2 IgG polyclonal antibody (dilution 1 : 100; Cell Signaling Technology), APC-conjugated rat antibody anti-mouse CD45 (BD, clone 30-F11), and PE-conjugated rat antibody anti-mouse CD64 (BD, Clone X54-5/7.1) diluted in 1% GS with 0.05% Tween-20. On the next day, samples were washed and incubated with anti-rabbit antibodies conjugated with Alexa Fluor 488 or Alexa Fluor 647 (dilution 1 : 1000; IgG H+L, Life Technologies) for 1 h at RT. Nuclei were counterstained with Hoechst 33342 (1 *μ*g/mL, Sigma-Aldrich) during the second washing. Negative controls are presented in Fig. S7. Samples were analysed under a fluorescent microscope (Nikon Eclipse TE 2000-U microscope fitted with a camera) at magnifications of 100x, 200x, and 400x or a meta laser scanning confocal microscope (LSM-880; Carl Zeiss) and analysed using ImageJ software (Wayne-Rasband (NIH)).

### 2.10. *In Situ* Gelatin Zymography (ISZ) with MMP-9 Analysis

*In situ* gelatinolytic activity in the media of a frozen abdominal aorta was performed as described previously [13]. A 1 mg/mL stock solution of fluorescein-conjugated, dye-quenched gelatin from a pig skin (DQ™-gelatin, Thermo Fisher Scientific) was prepared in gelatinase reaction buffer (150 mM NaCl, 5 mM CaCl_2_, 0.2 mM NaN_3_, 50 mM Tris-HCl, pH 7.6) and stored at 4°C. The working solution for DQ-gelatin was made by directly diluting DQ-gelatin stock solution in reaction buffer to a final concentration of 20 *μ*g/mL. Frozen, unfixed 8 *μ*m sections were thawed, marked with PAP pen, covered with DQ-gelatin working solution, and incubated at 37°C in a dark wet chamber for 2 h. Next, slides were washed three times in MilliQ water, fixed for 1 minute in ice-cold acetone, washed three times in PBS, and blocked in the wet, dark chamber with 10% GS with 0.05% Tween-20 for 1 h at RT. Further, scraps were overlaid with rabbit anti-mouse MMP-9 polyclonal antibodies (1 : 100, Cell Signaling Technology) in 1% GS with 0.05% Tween-20 and left overnight at 4°C in a dark wet chamber. On the next day, samples were washed and incubated with anti-rabbit antibodies conjugated with Alexa Fluor 568 for collagens I and III (dilution 1 : 1000; IgG H+L, Life Technologies) for 1 h at RT. Nuclei were counterstained with Hoechst 33342 (1 *μ*g/mL, Sigma-Aldrich) during the second washing. Samples were mounted in DAKO mounting medium and analysed under a fluorescent microscope (Nikon Eclipse TE 2000-U microscope fitted with a camera) at magnification 100x and analysed using ImageJ software.

The specificity of the ISZ protocol was confirmed through negative controls. Samples were preincubated for 1 h at 37°C with 1 mM 1,10-phenanthroline (gelatinase activity blockage) dissolved in gelatinase reaction buffer, then washed with PBS and incubated with the working solution for DQ-gelatin and stained for MMP-9 as described above (negative controls are presented in Fig. S6B). Finally, inhibition of DQ-gelatinase activity was analysed under a fluorescent microscope (Nikon Eclipse TE 2000-U microscope fitted with a camera) at a magnification of 100x and analysed using ImageJ software.

### 2.11. Human Abdominal Aortic Aneurysm Samples

Detailed characteristics of patients are described in our previous papers [14, 15]. Aneurysm wall tissue was collected during surgery. AAA diameter was measured with preoperative computed tomography angiography. The study was approved by the local institutional ethics committee (EC 294/2009) at the Medical University of Vienna.

### 2.12. Statistical Analysis

Data are presented as the mean ± SEM. Three-way ANOVA, followed by Tukey's post hoc test, was used for the comparison of more than two groups. Fisher's exact test was used to calculate the frequency of aneurysm appearance and rupture. Spearman's test was applied to calculate correlations. Grubbs' test was used to detect statistically significant outliers (*p* < 0.05), which were not included in the statistical analysis of the results (GraphPad Prism software). *p* < 0.05 was accepted as statistically significant.

## 3. Results

### 3.1. Simvastatin Reduces the Risk of AAA Formation and Rupture in Mice Lacking the Functional Nrf2

**Figure 1:**
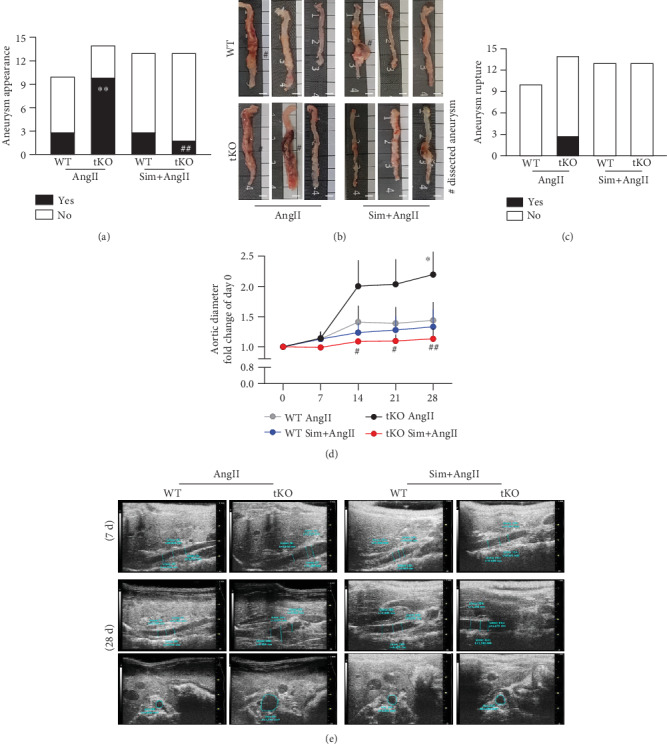
Simvastatin reduces the risk of AAA formation and rupture in Nrf2 tKO mice. Mice of both genotypes were divided into the following groups: (1) sham (saline, *n* = 8), (2) angiotensin II (AngII group, *n* = 10 or 14), (3) simvastatin+saline (Sim group, *n* = 12), and (4) simvastatin+angiotensin II (Sim+AngII group, *n* = 13). Simvastatin was administered daily for 7 consecutive days before osmotic pump placement and during AngII infusion for another 28 days. (a) The frequency of aortic aneurysm appearance (Fisher's exact test), and (b) representative image of aortas isolated from WT and Nrf2 tKO mice. Hashtag indicates a dissected aneurysm. Scale bar = 4 mm. (c) The frequency of aneurysm rupture (Fisher's exact test). (d) Time-dependent changes in the aortic inner diameter measured with USG. Three-way ANOVA and Tukey's post hoc test. (e) Representative images of aortic inner diameter changes in WT and Nrf2 tKO mice at days 7 and 28 of AngII and Sim+AngII treatment ^∗^*p* < 0.05 and ^∗∗^*p* < 0.01 vs. saline; ^#^*p* < 0.05 and ^##^*p* < 0.01 vs. AngII.

The classical model of AAA induction is based on AngII infusion in ApoE knockout (ApoE KO) mice, which lead to 70-80% aneurysm incidence [16]. However, given the debatable role of hyperlipidaemia in aneurysm etiology [17, 18] and possible aggravation of aneurysm formation under Nrf2 transcriptional deficiency, we decided to use a model of AngII-induced AAA combined with fat-enriched diet in C57BL6/J mice, instead of the ApoE KO mice. This model provided the formation of AAA in 30% of the animals (Figures 1(a) and 1(b)). In response to AngII infusion, none of the animals exhibited a dilatation in the thoracic or ascending part of the aorta, independently of the abdominal region. To analyse the effect of Nrf2 transcriptional activity, we used C57BL6/J mice with a transcriptionally inactive form of Nrf2 (Nrf2 tKO). Such mice were significantly more susceptible to aneurysm formation in comparison to WT counterparts since AngII infusion resulted in a 3-fold increase in the frequency of AAA (Figures 1(a) and 1(b)). Moreover, AAA rupture was observed in Nrf2 tKO animals only and occurred up to day 6 after AngII infusion (Figure 1(c)). The AAA formation was reduced by 80% by simvastatin in Nrf2 tKO mice (Figures 1(a) and 1(b)), and sudden death caused by the aneurysm rupture was also abolished (3/14 vs. 0/11 in AngII vs. Sim+AngII groups; Figure 1(c)). No significant morphological changes were observed in the aortas of the saline- or Sim-treated groups (Fig. S4A, B).

In contrast to WT mice, AngII infusion significantly increased the maximal aortic diameter in Nrf2 tKO animals, which was measured with ultrasonography (USG) at the suprarenal region of the abdominal aorta (Figures 1(d) and 1(e)). An initial aortic dilatation was captured on USG in the longitudinal plane as early as day 7 in AngII-treated Nrf2 tKO mice. Such an early dilatation was commonly associated with further dissection of the aorta at day 28 (longitudinal and transverse plane; Figure 1(e)). These effects were counteracted by simvastatin treatment (Figures 1(d) and 1(e)).

### 3.2. Simvastatin Delays the AngII-Induced Blood Pressure Rise

**Figure 2:**
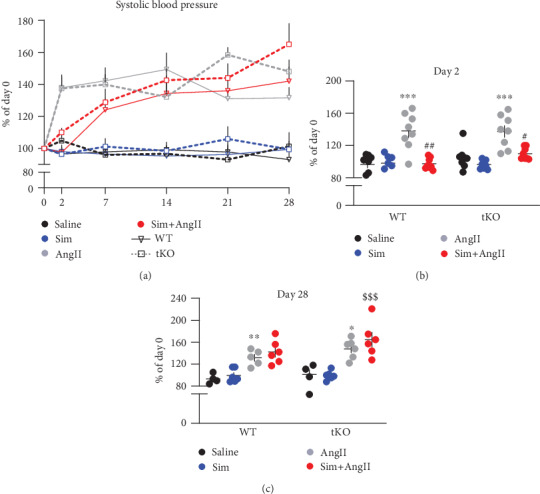
Simvastatin delays AngII-induced abrupt blood pressure rise. Mice of both genotypes were divided into the following groups: (1) sham (saline; *n* = 8), (2) angiotensin II (AngII group, *n* = 10 or 14), (3) simvastatin+saline (Sim group, *n* = 11), and (4) simvastatin+angiotensin II (Sim+AngII group, *n* = 13). Simvastatin was administered daily for 7 consecutive days before osmotic pump placement and during AngII infusion for another 28 days. (a) Time course of changes in systolic blood pressure (SBP) in WT and Nrf2 tKO, presented as a change of day 0. (b) SBP at day 2, presented as a change of day 0. Three-way ANOVA and Tukey's post hoc test. (c) SBP at day 2, presented as a change of day 0. Three-way ANOVA and Tukey's post hoc test. ^∗^*p* < 0.05, ^∗∗^*p* < 0.01, and ^∗∗∗^*p* < 0.001 vs. saline; ^#^*p* < 0.05 and ^##^*p* < 0.01 vs. AngII; ^$$$^*p* < 0.001 vs. Sim.

AngII increases systemic blood pressure, which may lead to vascular damage. It also perpetuates inflammation and oxidative stress, both directly associated with the formation of AAA [2, 3, 19]. Therefore, we monitored the time course of blood pressure rise upon the AngII infusion. AngII caused an abrupt increase in systolic blood pressure (SBP) in mice of both genotypes within the first two days (34.5% in WT and 40.1% in Nrf2 tKO). However, whereas in WT animals the blood pressure stabilised at day 14, in Nrf2 tKO mice, it tended to rise until day 28. So, at the end of the experiment, SBP in the AngII-treated Nrf2 tKO mice was around 45.0% higher than that in the Nrf2 tKO sham group and over 15% higher in comparison to that in AngII-treated WT mice (Figures 2(a) and 2(b)).

Importantly, simvastatin attenuated the rise of SBP in both genotypes on day 2. In Sim+AngII WT mice, SBP rose gradually up to day 14 and then remained stable. In Sim+AngII-administered Nrf2 tKO mice, SBP did not stabilise and on day 28 was 65.0% higher compared to day 0 (Figures 2(a) and 2(b)). These effects of simvastatin referred only to its combination with AngII. Simvastatin alone did not influence SBP in any of the genotypes (Figures 2(a) and 2(b)).

### 3.3. AAA Formation in Nrf2 tKO Mice Is Accompanied by an Inflammatory Response

**Figure 3:**
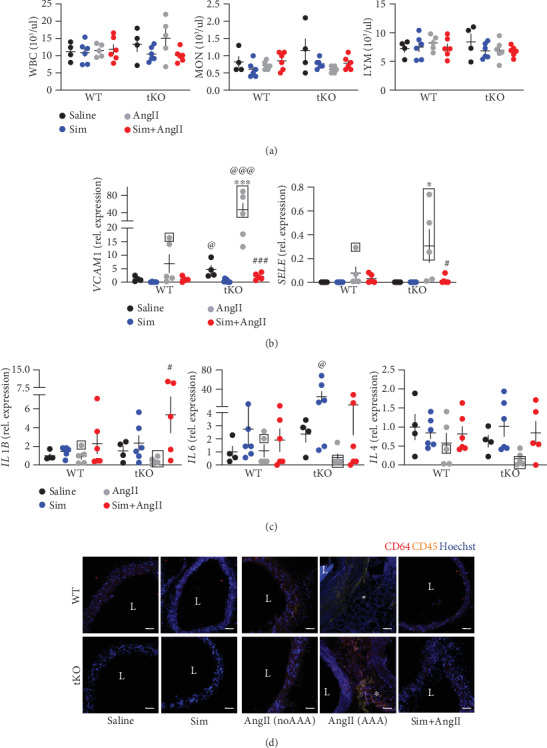
Immune cell infiltration and increased VCAM1 level are associated with AAA formation in Nrf2 tKO mice. Mice of both genotypes represent the following groups: (1) sham (saline, *n* = 4), (2) angiotensin II (AngII group, *n* = 5), (3) simvastatin+saline (Sim group, *n* = 6), and (4) simvastatin+angiotensin II (Sim+AngII group, *n* = 6). Simvastatin was administered daily for 7 consecutive days before osmotic pump placement and during AngII infusion for another 14 days. (a) Quantification of circulating white blood cells and their subpopulations (LYM: lymphocytes; MON: monocytes; GRA: granulocytes). Three-way ANOVA with Tukey's post hoc test. (b) Relative expression of *VCAM1* and *SELE* in the aortic wall. *eEF2* was used as a reference gene. Three-way ANOVA with Tukey's post hoc test. (c) Relative expression of *IL-1b*, *IL4*, and *IL6* in the aortic wall. *eEF2* was used as a reference gene. Three-way ANOVA with Tukey's post hoc test. Rectangle—mice which developed the aneurysm. (d) Immunofluorescent staining of infiltrating CD45^+^ and CD64^+^ cells within the aortic wall. CD45: yellow; CD64: red; nuclei: blue; L: lumen; magnification at 200x. Scale bar = 50 *μ*m. ^∗^*p* < 0.05 and ^∗∗∗^*p* < 0.001 vs. saline; ^#^*p* < 0.05 and ^###^*p* < 0.001 vs. AngII; ^@^*p* < 0.05 and ^@@@^*p* < 0.001 vs. WT.

One of the mechanisms of AngII-induced AAA development is the recruitment of monocytes and their differentiation to the effector macrophages [3]. This is why we assessed the inflammatory response to AngII infusion. The analysis of the count and profile of circulating leukocytes on day 14 revealed no differences between inspected groups (Figure 3(a). On the other hand, in the aortic tissue of Nrf2 tKO mice, AngII led to a remarkable upregulation of *VCAM1* (vascular cell adhesion molecule-1) and *SELE* (E-selectin) expression, genes coding for endothelial adhesion proteins that mediate the recruitment of leukocytes into sites of inflammation. This increase was the most pronounced in the individuals which have developed the aneurysms (Figure 3(b)). Of note, even in saline-treated Nrf2 tKO mice, the level of *VCAM1* was significantly higher than that in WT counterparts, implying the inflammatory state of intact Nrf2 tKO aortas (Figure 3(b)). AngII-induced increase in *VCAM1* and *SELE* expression was completely inhibited by simvastatin.

Expression of proinflammatory cytokines, such as *IL1B* (known to be expressed mainly in activated macrophages), *IL6* (which can be induced in monocytes and macrophages in response to IL1*β*), and *IL4* (known to be expressed primarily in lymphocytes), was detectable in the aortic tissue of both genotypes, but not significantly affected by AngII. Simvastatin showed a tendency to increase the expression of cytokines in Nrf2 tKO aortas (Figure 3(c)). The infiltration of CD64^+^/CD45^+^ myeloid cells into the aortic wall was present only in those animals that have developed the aneurysms upon AngII infusion and seemed to be more pronounced in Nrf2 tKO mice. Simvastatin attenuated the infiltration of myeloid cells in mice of both genotypes (Figure 3(d)).

Changes in circulating leukocyte content and expression of endothelial adhesion molecules were all attenuated on day 28 (Fig. S5A, B). Therefore in the next analyses, we focused on day 14, as a selected time point.

### 3.4. AngII-Induced Oxidative Stress Is Not Affected by Simvastatin

**Figure 4:**
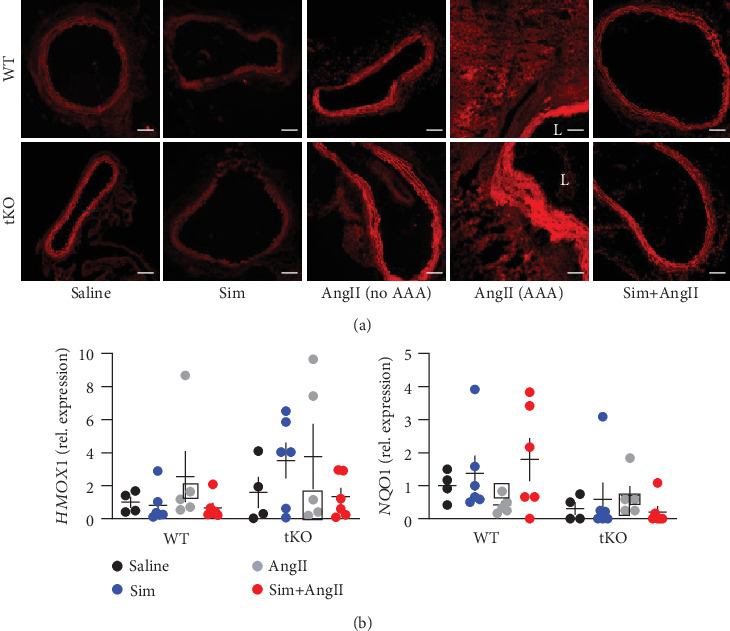
AngII-induced ROS level is not reduced by simvastatin. Mice of both genotypes represent the following groups: (1) sham (saline, *n* = 4), (2) angiotensin II (AngII group, *n* = 5), (3) simvastatin+saline (Sim group, *n* = 6), and (4) simvastatin+angiotensin II (Sim+AngII group, *n* = 6). Simvastatin was administered daily for 7 consecutive days before osmotic pump placement and during AngII infusion for another 14 days. (a) Assessment of ROS level in the abdominal aortic tissue. Representative pictures; L: lumen; magnification: 100x. Scale bar = 0.1 mm. (b) Relative expression of *HMOX-1* and *NQO1* in the abdominal aortic wall. *eEF2* was used as a reference gene. Three-way ANOVA with Tukey's post hoc test.

AAA aetiology strongly relies on oxidative stress [2, 3], and Nrf2 is the essential regulator of the cellular oxidative stress response [4]. Therefore, we evaluated the redox status of aortas. The level of reactive oxygen species (ROS) was assessed using a fluorescent probe. The comparison of control aortas from the sham WT and Nrf2 tKO mice revealed a modest increase in ROS in the Nrf2 tKO animals. AngII increased the ROS production in WT aortas to the level seen in Nrf2 tKO mice (Figure 4(a)). Importantly, in animals that have developed AAA, the ROS level within the aneurysmal tissue was higher, independently of the genotype. Simvastatin did not attenuate the effect of AngII treatment, regardless of the mouse genotype. Both in the WT and in Nrf2 tKO animals, simvastatin alone or in combination with AngII did not change or had a minor effect on the expression of antioxidative genes, *HMOX1* (heme oxygenase-1) and *NQO1* (NAD(P)H quinone dehydrogenase-1) (Figure 4(b)).

### 3.5. Simvastatin Mitigates AngII-Dependent Aortic Wall Rearrangements

**Figure 5:**
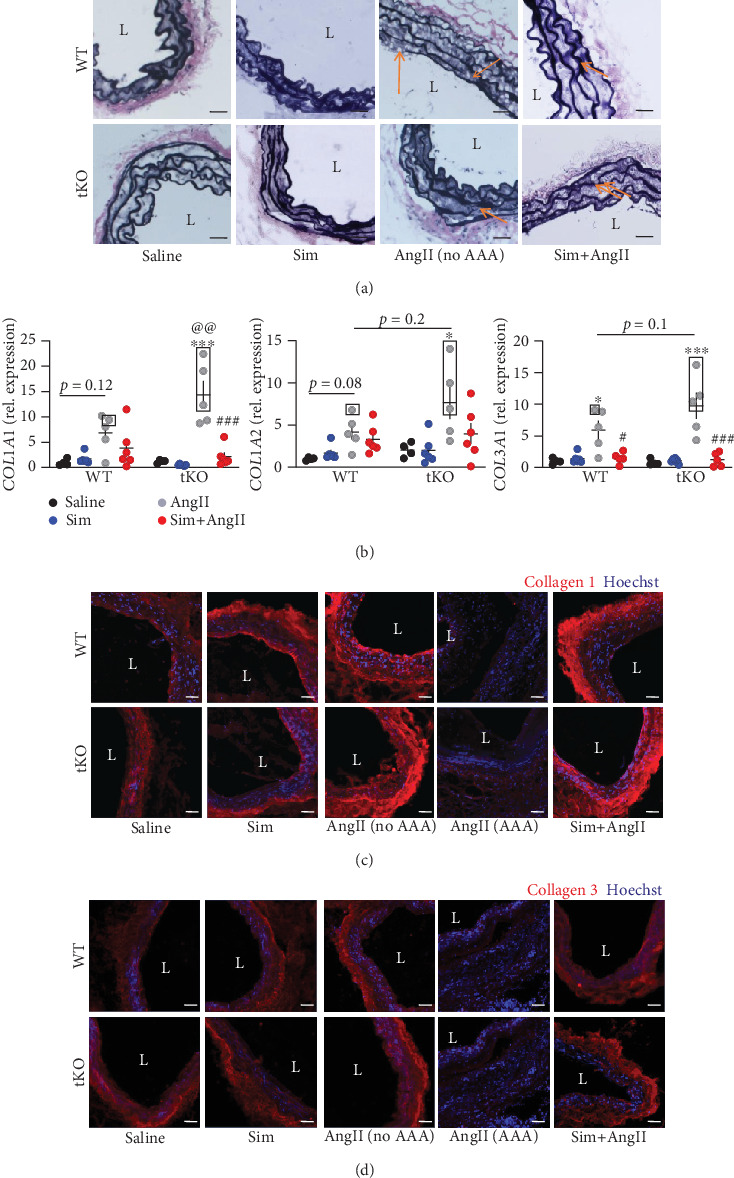
Simvastatin mitigates AngII-induced changes in collagen and elastin status. Mice of both genotypes represent the following groups: (1) sham (saline, *n* = 4), (2) angiotensin II (AngII group, *n* = 5), (3) simvastatin+saline (Sim group, *n* = 6), and (4) simvastatin+angiotensin II (Sim+AngII group, *n* = 6). Simvastatin was administered daily for 7 consecutive days before osmotic pump placement and during AngII infusion for another consecutive 14 days. (a) Verhoeff's Van Gieson staining of abdominal aortas for elastin fibers at magnification 200x. L: lumen. Scale bar = 25 *μ*m; arrow—elastin breaks. (b) Relative expression of *Col1α1*, *Col1α2*, and *Col3α1* within the abdominal aortic wall. *eEF2* was used as a reference gene. Three-way ANOVA with Tukey's post hoc test. Rectangle—mice, which developed the aneurysm. Immunofluorescent staining of (c) collagen I and (d) collagen III within the abdominal aorta at magnification 200x. L: lumen. Collagen: red; nuclei: blue. Representative images. Scale bar = 50 *μ*m. ^∗^*p* < 0.05 and ^∗∗∗^*p* < 0.001 vs. saline; ^#^*p* < 0.05 and ^###^*p* < 0.001 vs. AngII.

Formation of an aneurysm is strictly related to aortic wall rearrangements [2]. Elastin Van Gieson (EVG) staining confirmed the appearance of elastin damage in mice treated with AngII, regardless of genotype or aneurysm incidence. The occurrence of elastin damage was partially attenuated by simvastatin (Figure 5(a)). AngII also promoted collagen transcription (*COL1A1*, *COL1A2*, and *COL3A1*), the effect more pronounced in Nrf2 tKO mice and intensified in mice that have developed an aneurysm (Figure 5(b)). It was followed by changes in immunofluorescent staining of collagen I, but not collagen III. Simvastatin reduced the AngII-mediated increase in *COL1A1* and *COL3A1* expression (Figure 5(b)), but this effect was not visible at the protein level (Figure 5(c)).

Interestingly, a significant reduction in collagen I and III content was observed in the aneurysmal tissue, suggesting the enhanced protein degradation (Figure 5(c)). High collagen degradation within the aortic wall could be explained by the enhanced MMP (matrix metalloproteinase) activity. Hence, we performed *in situ* zymography (ISZ) and costained samples for MMP9, which is known to be strongly expressed in AAA tissue [20].

**Figure 6:**
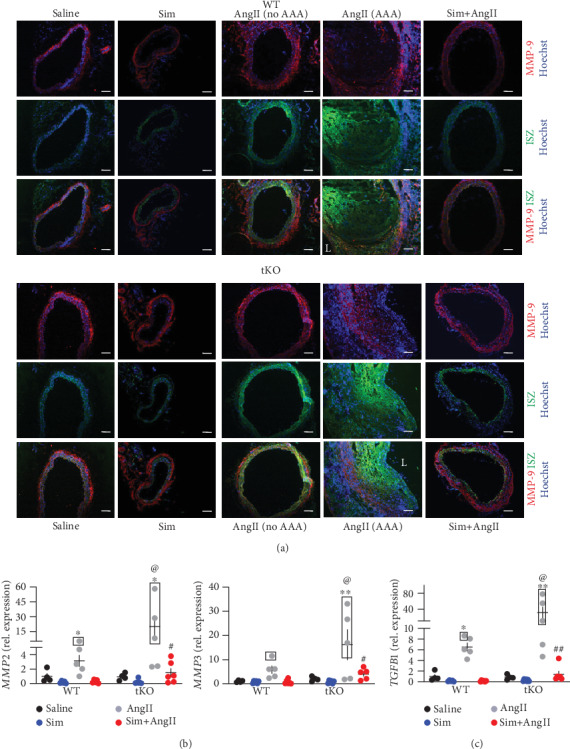
AAA formation is associated with enhanced metalloproteinase activity. Mice of both genotypes represent the following groups: (1) sham (saline, *n* = 4), (2) angiotensin II (AngII group, *n* = 5), (3) simvastatin+saline (Sim group, *n* = 6), and (4) simvastatin+angiotensin II (Sim+AngII group, *n* = 6). Simvastatin was administered daily for 7 consecutive days before osmotic pump placement and during AngII infusion for another 14 days. (a) The activity of gelatinases (green) and MMP-9 level (red) at a magnification of 100x. Blue: nuclei. The activity of gelatinases was assessed by *in situ* zymography. Representative images. Scale bar = 0.1 mm. L: lumen. (b) Relative expression of *MMP2* and *MMP3* within the abdominal aortic wall. *eEF2* was used as a reference gene. Three-way ANOVA with Tukey's post hoc test. Rectangle—mice, which developed the aneurysm. (c) Relative expression of *TGFB1* within the abdominal aortic wall. *eEF2* was used as a reference gene. Three-way ANOVA with Tukey's post hoc test. Rectangle—mice, which developed the aneurysm. ^∗^*p* < 0.05 and ^∗∗^*p* < 0.01 vs. saline; ^#^*p* < 0.05 and ^##^*p* < 0.01 vs. AngII; ^@^*p* < 0.05 vs. WT.

AngII increased gelatinase activity, which was attenuated by simvastatin (Figure 6(a)). The increase was independent of MMP9 level (Figure 6(a)), although in Nrf2 tKO mice without aneurysms, MMP9 signal could colocalise with gelatinase activity, suggesting a role of MMP9 in aortic wall degradation. The activity of gelatinases was the highest within the tissue of dissecting aneurysms, but here, no colocalisation with MMP9 was observed (Figure 6(a)). In accordance with immunofluorescent staining, no significant changes were observed on the MMP9 mRNA level (Fig. S6A).

In the next step, we inspected the expression of other MMPs, *MMP2* and *MMP3*, known to play a role in aortic wall degradation during aneurysm formation [20]. AngII infusion promoted the expression of both *MMP2* and *MMP3*, and the effect was more pronounced in Nrf2 tKO mice. AngII-induced elevation of *MMP2* and *MMP3* transcripts was attenuated by simvastatin (Figure 6(b)). Of note, their pattern of expression was highly similar to the metalloproteinase activity (Figures 6(a) and 6(b)).

### 3.6. Simvastatin May Inhibit AT1R-Dependent Intracellular Signalling

AngII regulates vascular tension and blood pressure acting on its receptors (AT1R and AT2R) localised on endothelial and smooth muscle cells and influencing intercellular signalling, which plays a crucial role in aortic wall rearrangements [19]. AngII often acts in synergy with TGF*β* [21]. We inspected the expression of *TGFb1*, which showed a similar pattern of expression of *MMP2* and *MMP3*, but also *COL1A1*, *COL1A2*, and *COL3A1* (Figures 6(c), 6(b), and 5(b)).

**Figure 7:**
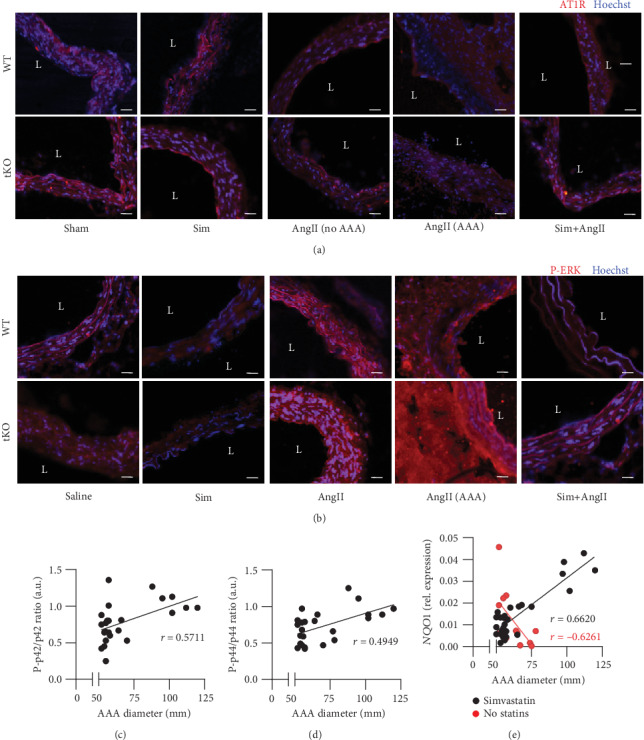
AT1R-dependent signalling upon AngII and simvastatin treatment. Mice of both genotypes represent the following groups: (1) sham (saline, *n* = 4), (2) angiotensin II (AngII group, *n* = 5), (3) simvastatin+saline (Sim group, *n* = 6), and (4) simvastatin+angiotensin II (Sim+AngII group, *n* = 6). Simvastatin was administered daily for 7 consecutive days before osmotic pump placement and during AngII infusion for another 14 days. Immunofluorescent staining of (a) AT1R and (b) P-ERK within the abdominal aortas at magnification 200x. L: aortic lumen. Representative images. Scale bar = 50 *μ*m. Correlation of AAA diameter with (c) P-p42/p42 ratio (Spearman's correlation *r* = 0.5711, *p* < 0.01, *n* = 24) and (d) P-p44/p44 ratio (Spearman's correlation *r* = 0.4949, *p* < 0.01, *n* = 24) and (e) correlation of AAA diameter with *NQO1* mRNA level among simvastatin-administered patients (black, Spearman's correlation *r* = 0.6620, *p* < 0.001, *n* = 28) and nonstatin-treated patients (red, Spearman's correlation *r* = 0.−6261, *p* < 0.05, *n* = 13).

The protein level of AT1R receptor remained unchanged irrespectively of the treatment (Figure 7(a)). However, considering the role of simvastatin in the inhibition of mevalonate pathways, thus influencing anchoring of signal transducers [22], we supposed that it might inhibit intracellular signalling from AT1R. We examined the activation of ERK1/2, which is one of the executors of downstream signalling from AT1R [19] and could partially serve as an indicator of pathway activation. The immunofluorescent staining confirmed the strong activation of ERK1/2, more pronounced in Nrf2 tKO mice infused with AngII in comparison to WT counterparts, which was abolished when mice were administered with simvastatin (Figure 7(b)). It might support the supposition that the protective effect of simvastatin in the prevention of aneurysm formation could be attributed to inhibition of AT1R intracellular signalling.

Then, we inspected the relationship between activation of ERK within aneurysm tissue in respect to its size, which reflects the progression of the aneurysm and the risk of its rupture. The characteristics of patients with AAA subjected to the analysis were described in our previous papers [14, 15]. AAA size positively correlated with the level of activated p42 (ERK1) (Figure 7(c), Spearman's correlation *r* = 0.5711, *p* < 0.01, *n* = 24) and activated p44 (ERK2) (Figure 7(d), Spearman's correlation *r* = 0.4949, *p* < 0.01, *n* = 24). Finally, we correlated AAA size with the level of Nrf2 transcriptional activity, assessed by expression of Nrf2 target gene *NQO1*. It revealed a positive correlation between aneurysm size and NQO1 level among patients administered with simvastatin (Figure 7(e)—black, Spearman's correlation *r* = 0.6620, *p* < 0.001, *n* = 28). On the contrary, among patients who did not receive statins, Nrf2 transcriptional activity negatively correlated with aneurysm susceptibility (Figure 7(e)—red, Spearman's correlation *r* = 0.−6261, *p* < 0.05, *n* = 13).

## 4. Discussion

Several studies discuss the role of Nrf2 in the regulation of not only oxidative stress but also vascular tone and thromboresistance [5, 23]. The proper functioning of those processes is crucial for the maintenance of vascular response to microinjuries which may forerun activation of MMPs, rearrangement of the extracellular matrix, and damage of elastin fibers that finally lead to the aortic stiffness, blood pressure upregulation, aortic dilatation, and aneurysm formation [2].

Here, we report that lack of transcriptionally active form of Nrf2 increases the risk of AAA development and rupture in mice. This matches the previous observation that activation of Nrf2 can counteract AAA formation [5] and confirms that Nrf2 plays a role in the prevention of AAA development. Our group showed that the aortas of Nrf2 tKO mice undergo premature senescence [24], which could increase the susceptibility to aneurysm formation. In accordance, age-related decline in Nrf2 transcriptional activity in human correlates with the incidence of cardiovascular disorders, also AAA [5].

Repeatedly, the incidence of AAA is parallel to the occurrence of atherosclerosis. Still, debate arises if atherosclerosis triggers the aneurysm formation. In our experimental setup, we observed no signs of atherosclerotic plaque development despite the consumption of a fat-enriched diet. It stays in line with literature data, showing that during AngII infusion, atherosclerotic lesions form after day 28 [25]. Still, it may substantiate that AAA formation may be independent of atherosclerosis progression. However, lipid-lowering drugs are prescribed to patients with an aortic aneurysm.

The role of statins in AAA prevention and treatment remains ambiguous. Nevertheless, analogously to our experiments, statin administration is protective in counteracting aortic dilatation in Marfan syndrome [26]. Several studies point out that the protective effects of statins depend on Nrf2 transcriptional activity [27, 28]. However, in our experimental setup, statins were protective independently of Nrf2 activation. It stays in line with our recent finding that in response to simvastatin Nrf2 translocates to the nucleus but does not stimulate ARE-driven transcription [14]. Accordingly, our data suggest a more protective effect of statins among patients with lower Nrf2 transcriptional activity. However, it would require more detailed analysis.

Up to date, the universal model of aneurysm formation has been the infusion of AngII to ApoE KO mice. However, given the debatable role of hyperlipidaemia in aneurysm etiology [17, 18] and possible aggravation of aneurysm formation under Nrf2 transcriptional deficiency, in our study, we decided to replace ApoE KO mice with mice of normal lipid metabolism but fed them a fat-enriched diet. By using such a strategy, we aimed to establish an AAA model using mice, which have been used for years. It could give us insights in AAA formation mechanisms and relate our previous findings to the impact on cardiovascular disorders. Aortas of Nrf2 transcriptional knockout mice undergo premature senescence, exhibit massive protein S-nitrosation [24], and enhanced protein aggregation [29] which could partially explain the higher susceptibility to AngII-induced AAA formation.

AngII-based models promote inflammatory response and mobilisation of monocytes from the spleen in case of ApoE knockout mice, which was found at the day 3 [30]. It may explain why in our experimental setup, we did not observe any significant changes in circulating leukocytes at later time points. Nonetheless, the formation of the aneurysm was associated with a higher inflammatory response, as evidenced by a higher VCAM1 expression and substantial myeloid cell infiltration. Of note, Nrf2 regulates cellular inflammatory response [31]. The loss of Nrf2 in macrophages enhances foam cell formation due to increased LDL (low-density lipoprotein) uptake and promotes the expression of the proinflammatory cytokines, e.g., MCP1, IL6, and TNF*α* [32]. In our experimental model, Nrf2 transcriptional deficiency did not affect the expression of IL1*β*, IL4, or IL6.

Looking for the mechanism of increased susceptibility to aneurysm onset, we monitored the blood pressure. Literature data indicate that Nrf2 deficiency contradicts hypertension by interference with angiotensin II metabolism and angiotensin receptor expression in diabetic mice [33]. Furthermore, Nrf2 regulates the activity of neurons that reside in the cardiovascular centre (the rostral ventrolateral medulla (RVLM)) by controlling the degradation of free radicals which cause sympathoexcitation of neurons and eventually hypertension in rodents [34]. In our study, up to day 14, i.e., during initial aneurysm development, the time course of changes in blood pressure was comparable between both genotypes. Data presented by Li et al. indicate that AngII infusion leads to a similar elevation in blood pressure in WT and Nrf2 tKO mice leading, however, to cardiac hypertrophy only in the latter [35]. That is why, considering the effect of simvastatin on AAA prevention and the fact that it attenuates the blood hypertension, we suppose that the abrupt blood pressure rise may lead to more severe damage in Nrf2 tKO aortas and trigger aneurysm formation. Of note, the hypotensive effect of statins is well documented [36], recurrently considered a side effect of statin-based therapy. In our experimental setup, however, it seems to have a protective outcome, possibly due to subsequent, rather than antecedent, AngII administration.

Aneurysm formation correlates with increased oxidative stress [2] or imbalance between key antioxidant players [37]. Although Nrf2 is a crucial regulator of cellular response to oxidative stress [4, 5], the inhibition of its transcriptional activity did not lead to excessive cellular ROS production in our animal model. It stays in agreement with our previous data that in the aortas of Nrf2 tKO mice, there are no signs of oxidative damage, manifested by lipid peroxidation [24]. Here, we demonstrated that irrespective of the genotype, AngII infusion led to an increase in the ROS level, which was partially attenuated by simvastatin treatment. Accordingly, we have shown that patients who were using simvastatin had a higher level of reduced glutathione [14]. Moreover, we observed a scarce effect on the key transcriptional targets of Nrf2, NQO1, and HMOX1. It is possible that under Nrf2 transcriptional deficiency, other transcription factors compensate for lack of Nrf2, such as AP family members [38]. It is also worth mentioning that Nrf2 possesses several noncanonical functions, which reach beyond its transcriptional activity. It may serve as a protein restraining Keap1, and also in this manner, it may impact the cellular homeostasis (reviewed in more detail in [39]).

Increased collagen turnover underlies the pathogenesis of aneurysm [40]. Of note, our data show that simvastatin partially mitigated changes in the aortic wall of Nrf2 tKO mice by influencing collagen production and degradation. We suppose that increased collagen turnover results from the overactivity of metalloproteinases, possibly MMP2 and MMP3, both previously reported as crucial in the aneurysm formation [20, 41]. The pattern of expression of those MMPs, especially MMP3, reflects the pattern of the gelatinase activity in the aortic wall, which could imply their role in rearrangement of the aortic wall and susceptibility of Nrf2 tKO mice to AAA formation. Furthermore, statin treatment led to the rearrangement of collagen deposition in the aortic wall. It promoted an increase in collagen-1 in the adventitia, possibly produced by fibroblasts, which could promote vascular integrity and protects against rupture during the increased blood pressure [42].

The analysis of ERK1/2 activation, which may mediate AT1R signalling, suggests that simvastatin may inhibit AT1R signalling, which is responsible for inflammatory response and rearrangements within the aortic wall. This supposition is in line with our previous observations where the reduced activity of oxidative stress-related mediators such as NF*κ*B and ERK1/2 as well as inhibition of MMP9 was found in AAA patients after simvastatin administration [15, 43, 44]. Similarly, lower collagen deposition was observed for rosuvastatin in myocardial cells [45] and for atorvastatin in airway smooth muscle cells, where additional downregulation of MMP9, VEGF, NF*κ*B, and TGF*β*1 was demonstrated [46]. Going further, we confronted the results in mice with human aneurysmal tissue, which showed that ERK1/2 activation strongly correlates with aneurysm size in patients. It is highly plausible that in our model based on C57Bl/6 mice, the effect of statin was independent of lipid-lowering action and more related to inhibition of inflammation and intracellular AT1R signalling. However, more detailed analyses, including assessment of lipid profile and impact on protein modification (e.g., prenylation), are needed to fully elucidate this point.

**Figure 8:**
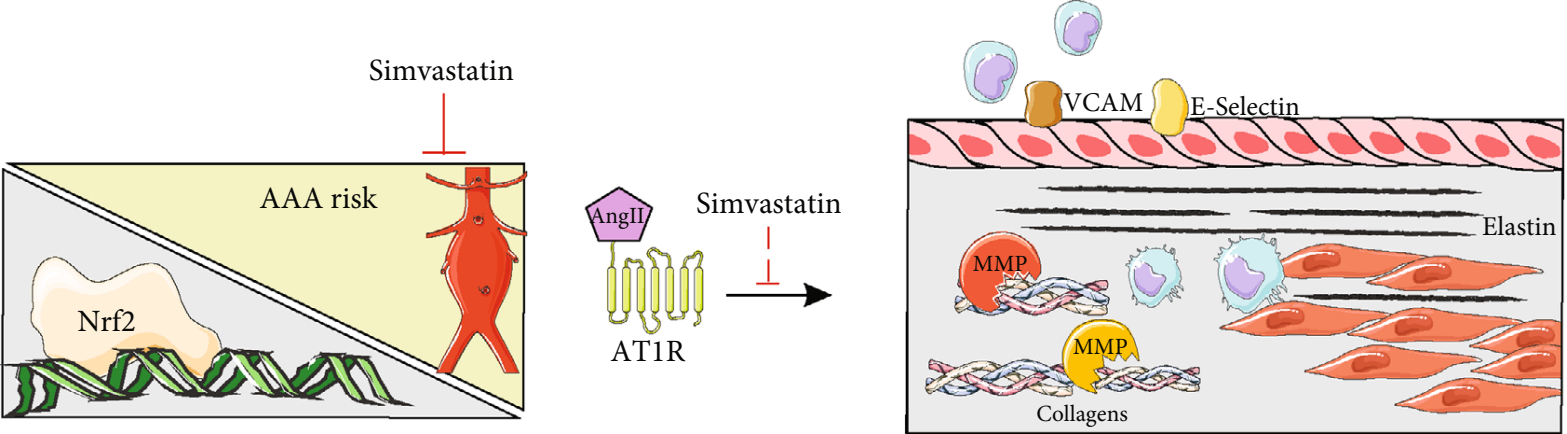
A graphical representation of the main findings of the article.

In conclusion, we show that lack of transcriptionally active form of Nrf2 increases the risk of AAA development and rupture in mice and that this process may be partially dependent on the vascular damage caused by an enhanced turnover of collagens and increased inflammatory response. These effects are mitigated by simvastatin administered before and during AngII infusion (Figure 8). Thus, it might be considered a potential prophylactic approach against AAA.

## Data Availability

The data used to support the findings of this study are available from the corresponding author upon request.

## Supplementary Materials

Supplementary Materials Supplementary Figure Legends: Fig. S1: confirmation of the model. Mice of both genotypes were divided into the following groups: (1) sham (saline, *n* = 8), (2) angiotensin II (AngII group; *n* = 10 or 14), (3) simvastatin+saline (Sim group; *n* = 12), and (4) simvastatin+angiotensin II (Sim+AngII group; *n* = 13). Simvastatin was administered daily for 7 consecutive days before osmotic pump placement and during AngII infusion for another 14 or 28 days. (A-B) Assessment of NFE2L2 mRNA level in the abdominal aorta. (A) Relative expression of NFE2L2 verified using primers recognizing the fragment, encoding the DNA binding domain (exon 5). eEF2 was used as a reference gene. Three-way ANOVA with Tukey's post hoc test. (B) Relative expression of NFE2L2 verified using primers recognizing the fragment, encoding the N terminal fragments of Nrf2, present in tKO mice (exon 3). eEF2 was used as a reference gene. Three-way ANOVA with Tukey's post hoc test. (C, D) Verification of statin administration through assessment of HMG-CoA reductase mRNA level. (C) Relative expression of HMGCR in the abdominal aorta. eEF2 was used as a reference gene. Three-way ANOVA with Tukey's post hoc test. (D) Relative expression of NFE2L2 verified using primers recognizing the fragment, encoding the N terminal fragments of Nrf2. Relative expression of HMGCR in the abdominal aorta. eEF2 was used as a reference gene. Three-way ANOVA with Tukey's post hoc test. ^**^*p* < 0.01 and ^***^*p* < 0.001 vs. saline; ^#^*p* < 0.05 and ^##^*p* < 0.01 vs. AngII; ^@@@^*p* < 0.001 vs. WT. Fig. S2: the scheme of simvastatin and angiotensin II dosage to WT and tKO mice together with time points of in vivo measurements of blood pressure (BP) and USG. Fig. S3: the schematic analysis of the aortic area and diameter from the B-mode (A) and M-mode (B) for representative mice at day 0 and day 28 after osmotic pump placement. MA: the mesenteric artery; LA: the left renal artery; RA: the right renal artery. Fig. S4: morphological and histological changes in the aorta of WT and tKO mice from control groups. (A) Representative image of aortas isolated from WT and tKO mice treated with saline and simvastatin+saline (Sim). Scale bar = 4 mm. (B) Time-dependent changes in the aortic inner diameter (mm) of control groups measured with USG. Three-way ANOVA with Tukey's post hoc test. ^**^*p* < 0.01 vs. saline. Fig. S5: the inflammatory response is attenuated at day 28. Mice of both genotypes were divided into the following groups: (1) sham (saline group, *n* = 4), (2) angiotensin II (AngII group, *n* = 5), (3) simvastatin+saline (Sim group, *n* = 6), (4) simvastatin+angiotensin II (Sim+AngII group, *n* = 7). Simvastatin was administered daily for 7 consecutive days before osmotic pump placement and during AngII infusion for another 28 days. (B) Relative expression of VCAM1 and SELE in the aortic wall. eEF2 was used as a reference gene. Three-way ANOVA with Tukey's post hoc test. ^*^*p* < 0.05 vs. saline. Fig. S6: supplementary information for the assessment of metalloproteinase activity. (A) Relative expression of MMP9 within the abdominal aortic wall. eEF2 was used as a reference gene. Three-way ANOVA with Tukey's post hoc test. Rectangle—mice, which developed the aneurysm. (B) Negative controls for in situ zymography (A) performed on the abdominal aorta in mice of both genotypes. Gelatinase activity was muted with 1 h incubation of aortic specimens with 1,10-phenanthroline (Phe, 10 *μ*M). Green-gelatinase activity, blue-nuclei. L: lumen. Scale bar = 0.1 mm. Fig. S7: negative controls for AT1R (A) and P-ERK (B) immunofluorescent stainings. L: lumen. Scale bar = 50 *μ*m. Supplementary Table 1: Table 1: the sequence of primers used in the study.
