# Supplementary material for: Simvastatin Attenuates Abdominal Aortic Aneurysm Formation Favoured by Lack of Nrf2 Transcriptional Activity
Source: Oxid Med Cell Longev. 2020 Jun 16;2020:6340190. doi: 10.1155/2020/6340190 (PMC7315306; doi:10.1155/2020/6340190)
Supplement: Supplementary Materials — Supplementary Figure Legends: Fig. S1: confirmation of the model. Mice of both genotypes were divided into the following groups: (1) sham (saline, n = 8), (2) angiotensin II (AngII group; n = 10 or 14), (3) simvastatin+saline (Sim group; n = 12), and (4) simvastatin+angiotensin II (Sim+AngII group; n = 13). Simvastatin was administered daily for 7 consecutive days before osmotic pump placement and during AngII infusion for another 14 or 28 days. (A-B) Assessment of NFE2L2 mRNA level in the abdominal aorta. (A) Relative expression of NFE2L2 verified using primers recognizing the fragment, encoding the DNA binding domain (exon 5). eEF2 was used as a reference gene. Three-way ANOVA with Tukey's post hoc test. (B) Relative expression of NFE2L2 verified using primers recognizing the fragment, encoding the N terminal fragments of Nrf2, present in tKO mice (exon 3). eEF2 was used as a reference gene. Three-way ANOVA with Tukey's post hoc test. (C, D) Verification of statin administration through assessment of HMG-CoA reductase mRNA level. (C) Relative expression of HMGCR in the abdominal aorta. eEF2 was used as a reference gene. Three-way ANOVA with Tukey's post hoc test. (D) Relative expression of NFE2L2 verified using primers recognizing the fragment, encoding the N terminal fragments of Nrf2. Relative expression of HMGCR in the abdominal aorta. eEF2 was used as a reference gene. Three-way ANOVA with Tukey's post hoc test. **p < 0.01 and ***p < 0.001 vs. saline; #p < 0.05 and ##p < 0.01 vs. AngII; @@@p < 0.001 vs. WT. Fig. S2: the scheme of simvastatin and angiotensin II dosage to WT and tKO mice together with time points of in vivo measurements of blood pressure (BP) and USG. Fig. S3: the schematic analysis of the aortic area and diameter from the B-mode (A) and M-mode (B) for representative mice at day 0 and day 28 after osmotic pump placement. MA: the mesenteric artery; LA: the left renal artery; RA: the right renal artery. Fig. S4: morphological and histological chan [file 6340190.f1.pdf]

Supplementary materials

Simvastatin attenuates abdominal aortic aneurysm formation favoured by lack of Nrf2 transcriptional activity

Aleksandra Kopacz[1], Ewa Werner[1,2], Anna Grochot-Przęczek[1], Damian Klóska[1], Karolina Hajduk[1], Christoph Neumayer[3], Alicja Józkowicz[1], Aleksandra Piechota-Polanczyk[1*]

[1]Department of Medical Biotechnology, Faculty of Biochemistry, Biophysics and Biotechnology, Jagiellonian University, Krakow, Poland.

[2]Department of Animal Reproduction and Anatomy, Faculty of Animal Science, University of Agriculture, Krakow, Poland

[3]Department of Surgery, Division of Vascular Surgery, Medical University of Vienna, Austria.

***Corresponding author:**

Aleksandra Piechota-Polanczyk, PhD, Department of Medical Biotechnology, Faculty of Biochemistry, Biophysics and Biotechnology, Jagiellonian University, Gronostajowa 7, 30-387 Krakow, Poland, e-mail: aleksandra.piechota-polanczyk@uj.edu.pl, phone: +48 12 664-64-12, Fax: +48 12 664-69-02

Supplementary Figure Legends:

Fig. S1

Confirmation of the model. Mice of both genotypes were divided into the following groups: 1) sham (saline, n=8); 2) angiotensin II (AngII group; n=10 or 14); 3) simvastatin + saline (Sim group; n=12); 4) simvastatin + angiotensin II (Sim+AngII group; n=13). Simvastatin was administered daily for 7 consecutive days before osmotic pump placement and during Ang II infusion for another 14 or 28 days. **(A-B)** Assessment of NFE2L2 mRNA level in the abdominal aorta**.** (**A**) Relative expression of *NFE2L2* verified using primers recognizing the fragment, encoding the DNA binding domain (exon 5). *eEF2* was used as a reference gene. 3-way ANOVA with Tukey's post hoc test. **(B**) Relative expression of *NFE2L2* verified using primers recognizing the fragment, encoding the N terminal fragments of Nrf2, present in tKO mice (exon 3). *eEF2* was used as a reference gene. 3-way ANOVA with Tukey's post hoc test.; (**C-D**) Verification of statin administration through assessment of HMG-CoA reductase mRNA level. (**C**) Relative expression of *HMGCR* in the abdominal aorta. *eEF2* was used as a reference gene. 3-way ANOVA with Tukey's post hoc test. **(D**) Relative expression of *NFE2L2* verified using primers recognizing the fragment, encoding the N terminal fragments of Nrf2). Relative expression of *HMGCR* in the abdominal aorta. *eEF2* was used as a reference gene. 3-way ANOVA with Tukey's post hoc test. $^{**}p<0.01$; $^{***}p<0.001$ vs saline; $^{\#}p<0.05$; $^{\#\#}p<0.01$ vs AngII, $^{@@@}p<0.001$ vs WT.

Fig. S2

The scheme of simvastatin and angiotensin II dosage to WT and tKO mice together with time points of *in vivo* measurements of blood pressure (BP) and USG.

Fig. S3

The schematic analysis of aortic area and diameter from the B-mode (A) **and M-mode** (B) for representative mice at day 0 and day 28 after osmotic pump placement. MA- the mesenteric artery; LA- the left renal artery; RA- the right renal artery.

Fig. S4

Morphological and histological changes in the aorta of WT and tKO mice from control groups. (A) Representative image of aortas isolated from WT and tKO mice treated with saline and simvastatin+saline (Sim). Scale bar = 4 mm (**B**) Time-dependent changes in the aortic inner diameter [mm] of control groups measured with USG. 3-way ANOVA with Tukey's post hoc test, $^{**}p<0.01$ vs saline.

Fig. S5

The inflammatory response is attenuated at day 28. Mice of both genotypes were divided into the following groups: 1) sham (saline group, (n=4); 2) angiotensin II (AngII group; n=5); 3) simvastatin + saline (Sim group; n=6); 4) simvastatin + angiotensin II (Sim+AngII group; n=7). Simvastatin was administered daily for 7 consecutive days before osmotic pump placement and during Ang II infusion for another 28 days. (**B**) Relative expression of *VCAM1, SELE* in the aortic wall. *eEF2* was used as a reference gene. 3-way ANOVA with Tukey's post hoc test. $^{*}p<0.05$ vs saline.

Fig. S6

Supplementary information for the assessment of metalloproteinase activity. (**A**) Relative expression of *MMP9* within the abdominal aortic wall. *eEF2* was used as a reference gene. 3-way ANOVA with Tukey's post hoc test. Rectangle – mice, which developed the aneurysm. (**B**) Negative controls for *in situ* zymography (Fig. 6A) performed on abdominal aorta in mice of both genotypes. Gelatinases activity was muted with 1 h incubation of aortic specimens with 1,10 phenanthroline (Phe, 10 μM). Green-gelatinases activity, Blue-nuclei. L – lumen. Scale bar 0.1 mm.

Fig. S7

Negative controls for AT1R (A) and P-ERK (B) immunofluorescent stainings (Fig. 7A-B). L – lumen. Scale bar 50 μm.

Supplementary Table 1

Table. 1 The sequence of primers used in the study.

Gene	Primer sequence
Mouse *Eef2*	Forward: 5’- GACATCACCAAGGGTGTGCA -3’<br>Reverse: 5’- TCAGCACACTGGCATAGACC -3’
Mouse *NFE2L2 (exon 3)*	Forward: CAGAGACATTCCCATTTGTAG<br>Reverse: ATTCGGGAATGGAAAATAGC
Mouse *NFE2L2 (exon 5)*	Forward: TCACACGAGATGAGCTTAGGGCAA<br>Reverse: TACAGTTCTGGGCGGCGACTTTAT
Mouse *HMGCR*	Forward: GATAGCTGATCCTTCTCCTC<br>Reverse: ATGCTGATCATCTTGGAGAG
Mouse *Vcam1*	Forward: CCGGCATATACGAGTGTGAA<br>Reverse: GATGCGCAGTAGAGTGCAAG
Mouse *Sele*	Forward: ATGCCTCGCGCTTTCTCTC<br>Reverse: GTAGTCCCGCTGACAGTATGC
Mouse *IL1β*	Forward: 5’- CTGGTGTGTGACGTTCCCATTA-3’<br>Reverse: 5’- CCGACAGCACGAGGCTTT -3’
Mouse *IL4*	Forward: 5’- CTGGTGTGTGACGTTCCCATTA -3’<br>Reverse: 5’- CCGACAGCACGAGGCTTT -3’
Mouse *IL6*	Forward: 5’-AAAGAGTTGTGCAATGCAATGGCAATTCT-3’<br>Reverse: 5’- AAGTGCATCATCGTTGTTCATACA -3’
Mouse *Hmox1*	Forward: 5’- TTCTTCACCTTCCCCAACATTG -3’<br>Reverse: 5’- CAGCTCCTGCAACTCCTCAAA -3’
Mouse *NQO1*	Forward: 5’-GCTCGTAGCAGGATTTTGCCT -3’<br>Reverse: 5’-CAGGATGCCACTTCTGAATCG -3’
Mouse *Col1α1*	Forward: 5’- ACTACCGGGCCGATGATGCTAACG -3’<br>Reverse: 5’- CGATCCAGTACTCTCCGCTCTTCC -3’
Mouse *Col1α2*	Forward: 5’- GCCACCATTGATAGTCTCTCC -3’<br>Reverse: 5’- CACCCCAGCGAAGAACTCATA -3’
Mouse *Col3α1*	Forward: 5’- ATCTATGAATGGTGGTTTTCAGTT -3’<br>Reverse: 5’- TTTTGCAGTGGTATGTAATGTTCT -3’
Mouse *MMP2*	Forward: 5’- ACAGGACATTGTCTTTGATG -3’

	Reverse: 5’- TACACAGCGTCAATCTTTTC – 3’
Mouse *MMP3*	Forward: 5’- CTAAAAGCATTCACACCCTG -3’<br>Reverse: 5’- TTTCTTCTCATCAAACCTCC -3’
Mouse *TGFβ1*	Forward: 5’-TGATAAAGTGGAGTGAAGAGAG -3’<br>Reverse: 5’-GGCTTTTTGTAGTTTCCTAGAG -3’

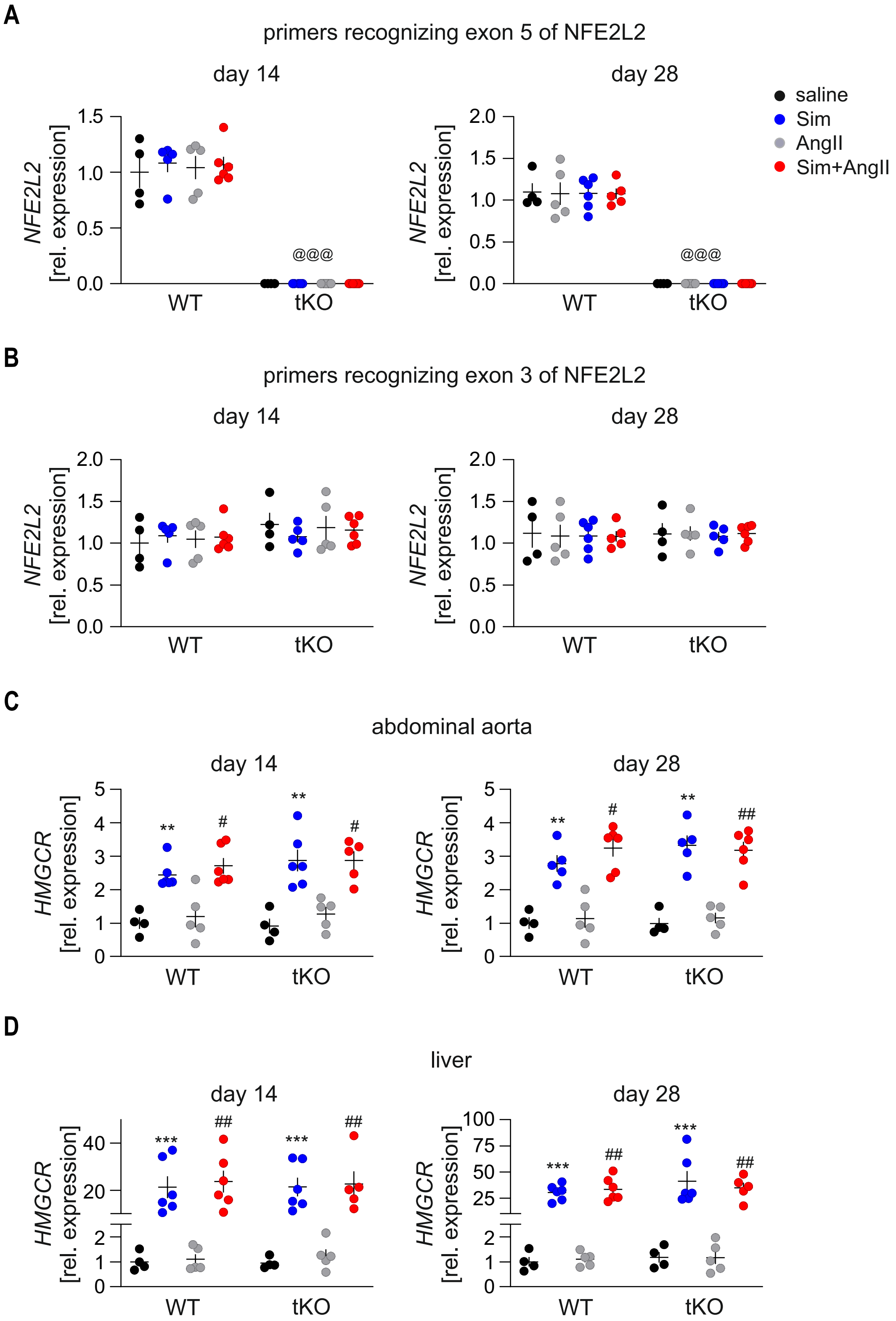
A
primers recognizing exon 5 of NFE2L2
day 14
day 28
saline
Sim
AngII
Sim+AngII
NFE2L2
[rel. expression]
1.5
1.0
0.5
0.0
2.0
@@@
WT
tKO
B
primers recognizing exon 3 of NFE2L2
C
abdominal aorta
HMGCR
5
4
3
2
1
0
**
#
##
D
liver
40
20
100
75
50
25
***

Fig. S1

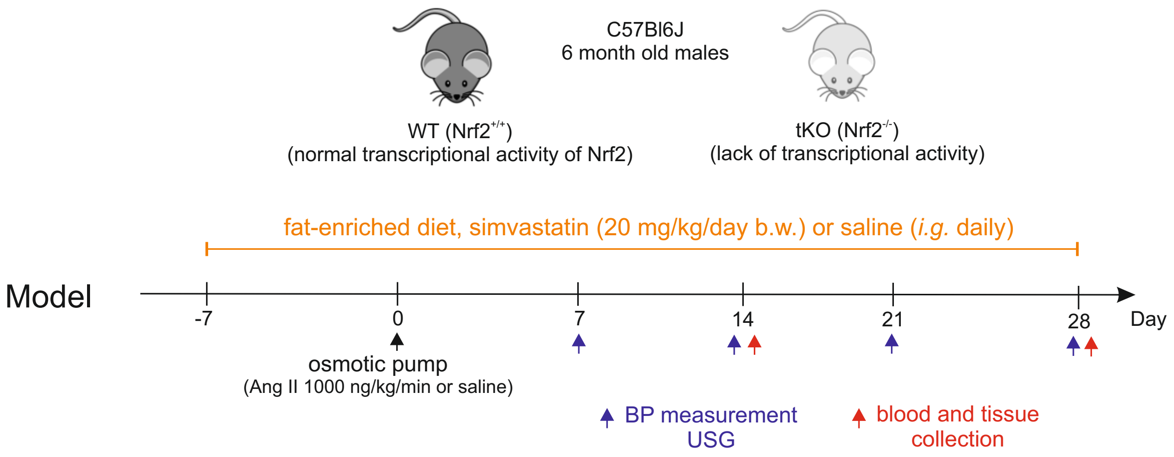
C57Bl6J
6 month old males
WT (Nrf2+/+)
(normal transcriptional activity of Nrf2)
tKO (Nrf2-/-)
(lack of transcriptional activity)
fat-enriched diet, simvastatin (20 mg/kg/day b.w.) or saline (i.g. daily)
Model
-7
0
7
14
21
28
Day
osmotic pump
(Ang II 1000 ng/kg/min or saline)
BP measurement
USG
blood and tissue
collection

Fig. S2

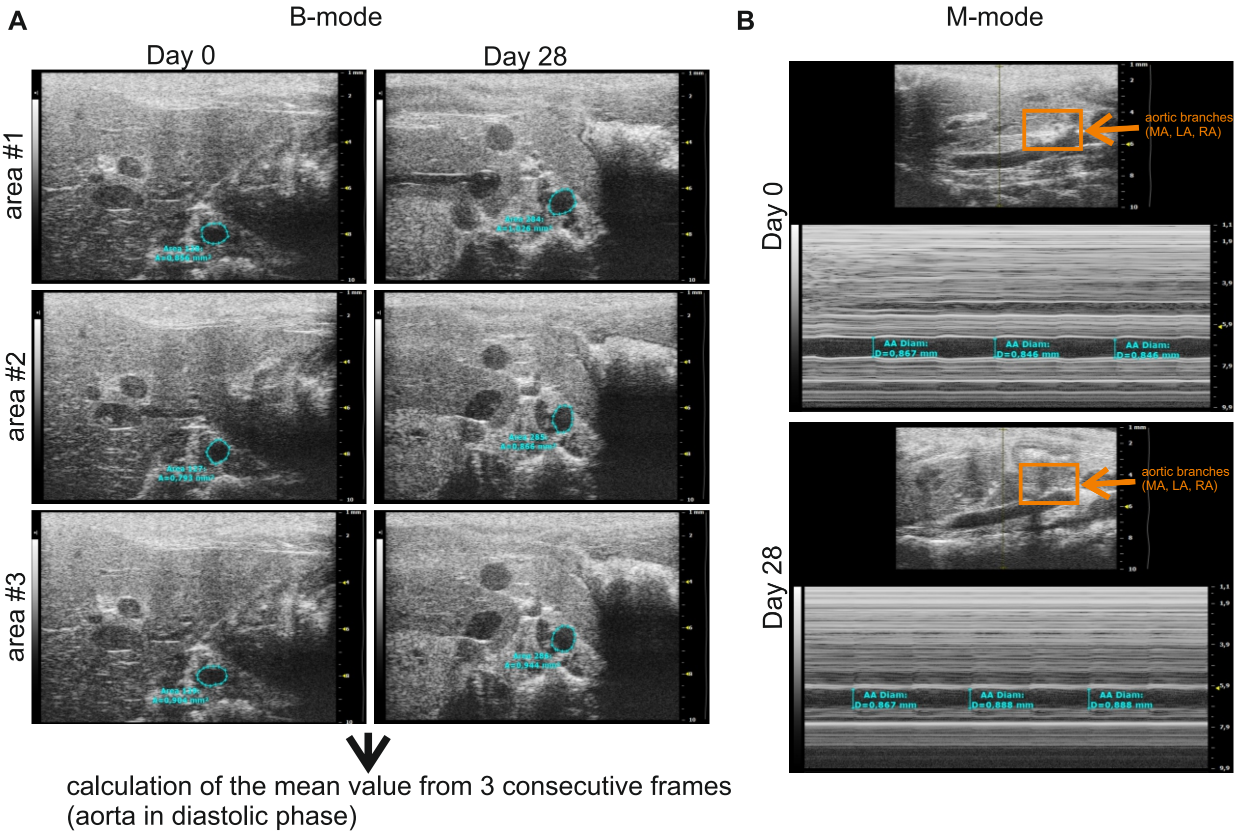
A
B-mode
Day 0
Day 28
area #1
area #2
area #3
calculation of the mean value from 3 consecutive frames
(aorta in diastolic phase)
B
M-mode
Day 0
Day 28
aortic branches
(MA, LA, RA)
aortic branches
(MA, LA, RA)
AA Diam:
D=0,867 mm
AA Diam:
D=0.846 mm
AA Diam:
D=0.846 mm
AA Diam:
D=0.867 mm
AA Diam:
D=0.888 mm
AA Diam:
D=0.888 mm

Fig. S3

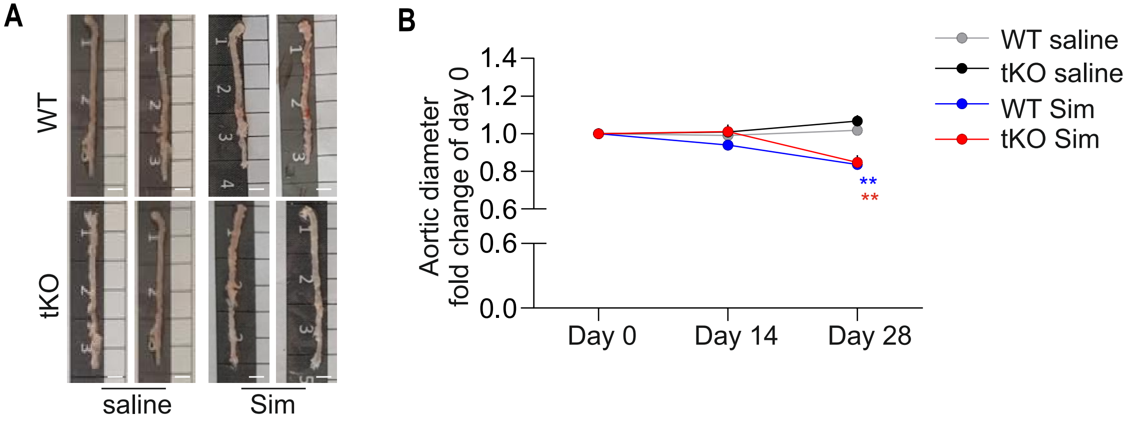
A
WT
tKO
saline
Sim
B
Aortic diameter
fold change of day 0
1.4
1.2
1.0
0.8
0.6
0.6
0.0
Day 0
Day 14
Day 28
**
**
WT saline
tKO saline
WT Sim
tKO Sim

Fig. S4

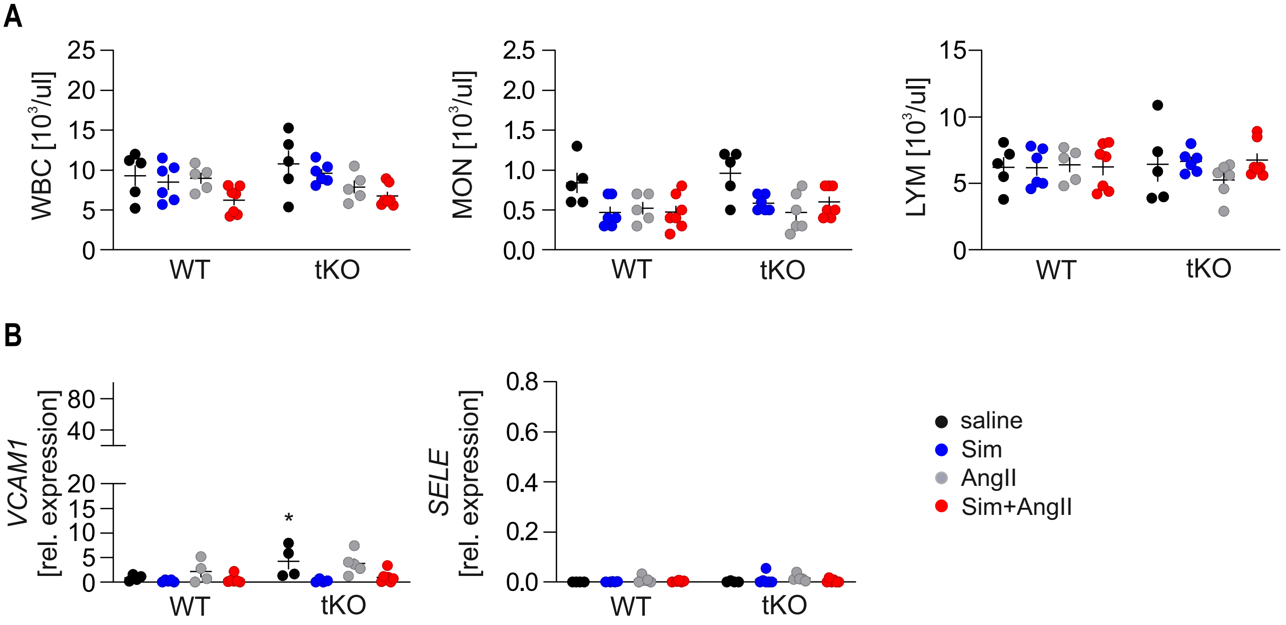

A
WBC [10³/ul]
MON [10³/ul]
LYM [10³/ul]
WT
tKO
B
VCAM1 [rel. expression]
SELE [rel. expression]
*
saline
Sim
AngII
Sim+AngII

Fig. S5

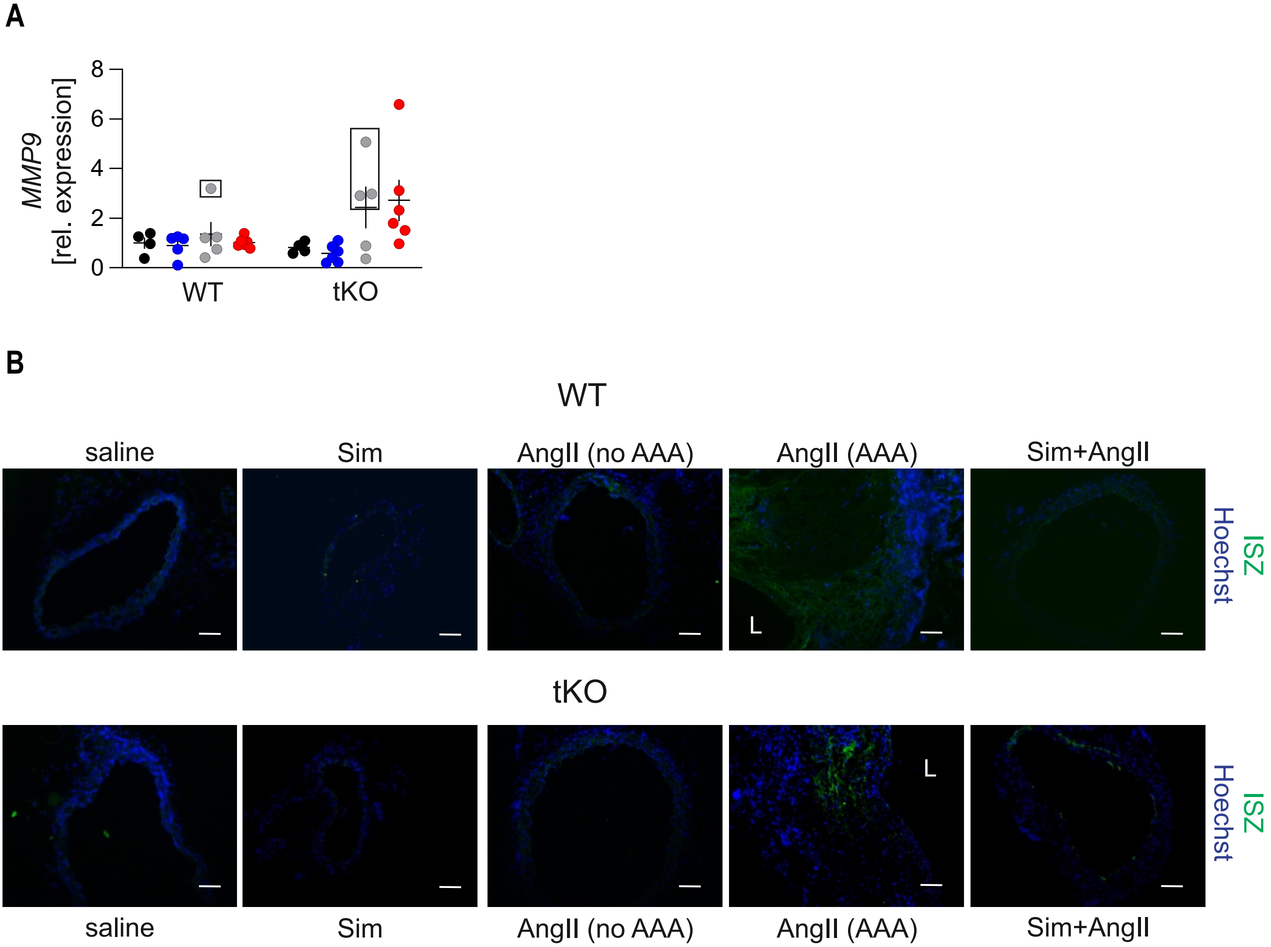
A
8
6
4
2
0
MMP9
[rel. expression]
WT
tKO
B
WT
saline
Sim
AngII (no AAA)
AngII (AAA)
Sim+AngII
L
ISZ
Hoechst
tKO
L
saline
Sim
AngII (no AAA)
AngII (AAA)
Sim+AngII
ISZ
Hoechst

Fig. S6

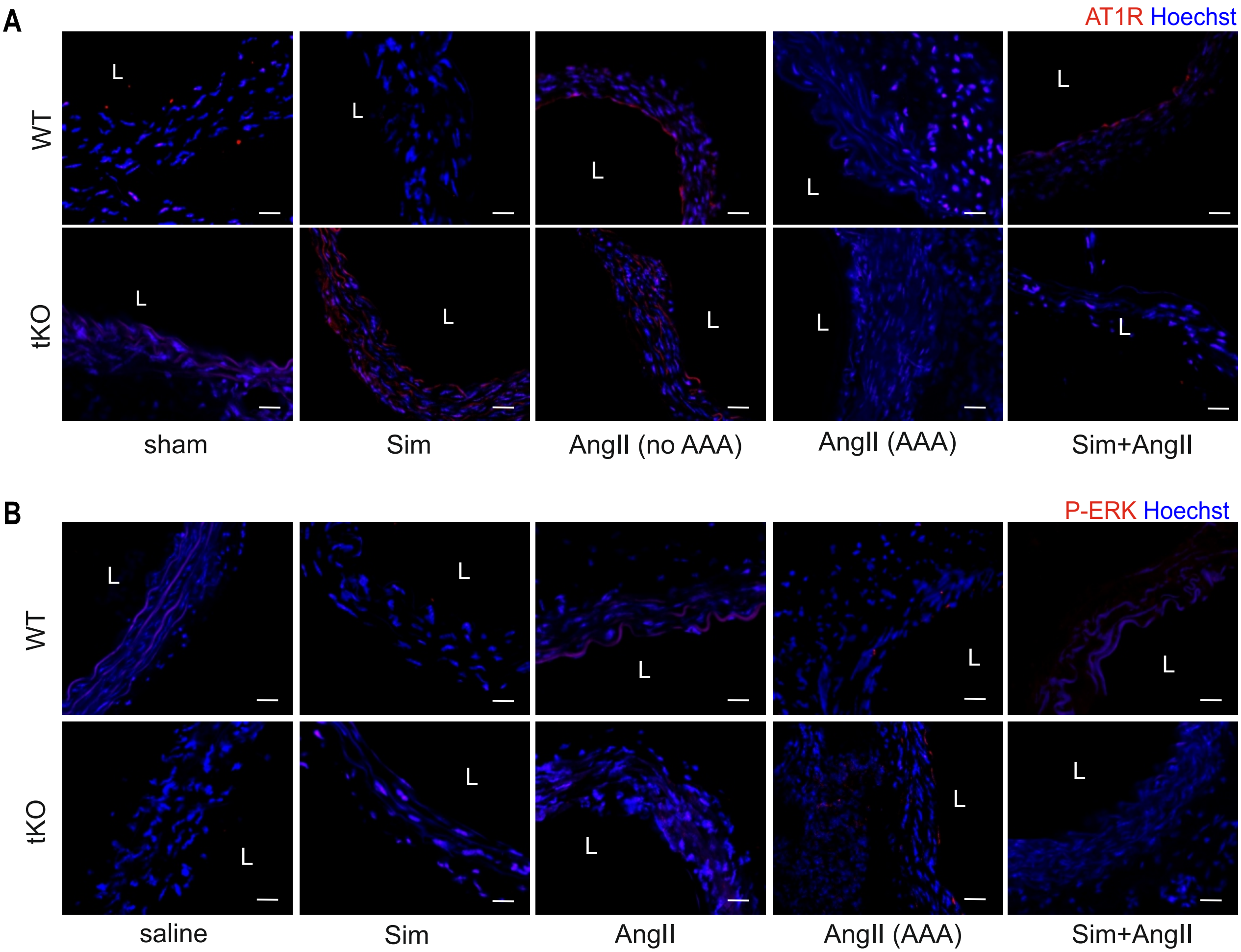
A
AT1R Hoechst
WT
tKO
L
sham
Sim
AngII (no AAA)
AngII (AAA)
Sim+AngII
B
P-ERK Hoechst
saline
Sim
AngII
AngII (AAA)
Sim+AngII

Fig. S7
